# Effects of anoxic prognostic model on immune microenvironment in pancreatic cancer

**DOI:** 10.1038/s41598-023-36413-9

**Published:** 2023-06-05

**Authors:** Yangdong Wu, Qingyan Kou, Lin Sun, Xiao Hu

**Affiliations:** 1 Department of Hepatobiliary Pancreatic Surgery, The Affiliated Hospital of Qingdao University, 16 Jiangsu Road, Qingdao, Shandong People’s Republic of China; 2 Department of ICU, The Affiliated Hospital of Qingdao University, Qingdao, China

**Keywords:** Biochemistry, Cancer, Immunology

## Abstract

Pancreatic cancer has one of the worst prognoses in the world, which suggests that the tumor microenvironment, which is characterized by hypoxia and immunosuppression, plays a significant role in the prognosis and progression of pancreatic cancer. We identified PLAU, LDHA, and PKM as key genes involved in pancreatic cancer hypoxia through GO/KEGG enrichment related hypoxia pathways and cox regression, established prognostic models, and studied their relationship to immune invasion through bioinformatics using R and related online databases. We verified the high expression of PLAU, LDHA, and PKM in pancreatic cancer cells using qPCR in vitro, and we also discovered that the expression of PLAU, LDHA, and PKM in hypoxic pancreatic cancer cells differed from that in normal cultured pancreatic cancer cells. Finally, we discovered that our prognostic model accurately predicted postrain in pancreatic cancer patients with hypoxia and immune infiltration.

## Introduction

The prognosis for pancreatic cancer is among the worst in the world, and it is the fourth leading cause of cancer-related death worldwide^1^.The specific position of the pancreas in the abdominal cavity causes concealed characteristics of pancreatic cancer in its early stages^2^, and the absence of extremely sensitive molecularly targeted markers for pancreatic cancer makes early diagnosis difficult^3,4^. Pancreatic cancer is highly aggressive^5^. Once diagnosed, it is likely to be advanced, making it difficult to find an effective treatment plan^5^. Although pancreatic cancer has been extensively studied in recent years and research results on its diagnosis, radiotherapy technology, and systematic treatment have been continuously proposed, the survival rate of the disease has not improved significantly, and the number of deaths related to the disease continues to rise^6^.

In pancreatic cancer, malignant cells account for only a small proportion; the remainder is composed primarily of fibroblasts, extracellular matrix, endothelial cells, and hematopoietic cells, and these host components constitute the tumor microenvironment^7^. The biological function of tumors is mainly determined by the interaction between cancer cells and their microenvironment^7,8^. These tumor microenvironment cell types contribute to a highly immunosuppressive, hypoxic, and pro-fibroproliferative cancer^7,9^. Hypoxia is one of the significant features of the pancreatic tumor microenvironment, which is due to a wide range of connective tissue hyperplasia and secondary vascular decrease^10^. Hypoxia is also one of the factors that contribute to the progression of pancreatic cancer^11^, and it has played a key role in a variety of cells and biological events, including cell proliferation, survival, angiogenesis, metabolism, tumor growth, invasion, and metastasis^12^. Hypoxia is one of the important microenvironmental characteristics of pancreatic cancer. Hypoxia microenvironment can induce HIF-1α factor list target to regulate downstream genes and promote downstream pathway activation.

Pancreatic cancer (PAAD) immune environment is generally considered immune suppression^13,14^. This immunosuppression is associated with poor prognosis with pancreatic cancer^15,16^, and the immune microenvironment of the tumor is very insensitive to immunotherapy, which causes a poor prognosis for pancreatic cancer^17^.

In conclusion, hypoxia and high immunosuppression in the microenvironment of pancreatic tumors play a significant role in the progression and prognosis of the tumor, and the development of relevant prognostic models is of great significance for the prognostic guidance of PAAD. At present, hypoxia is an essential factor in the pancreatic cancer microenvironment, and a model for predicting the prognosis of PAAD patients is an imperative necessity. The purpose of this study was to investigate special target molecules related to hypoxia in pancreatic cancer using bioinformatics methods, to investigate their role in the anoxic tumor microenvironment of pancreatic cancer, to establish related prognostic models, and to examine the important role of models in the immune microenvironment so as to provide new ideas for the prognosis and treatment of pancreatic cancer.

## Result

### Gene expression and enrichment analysis

**Figure 1:**
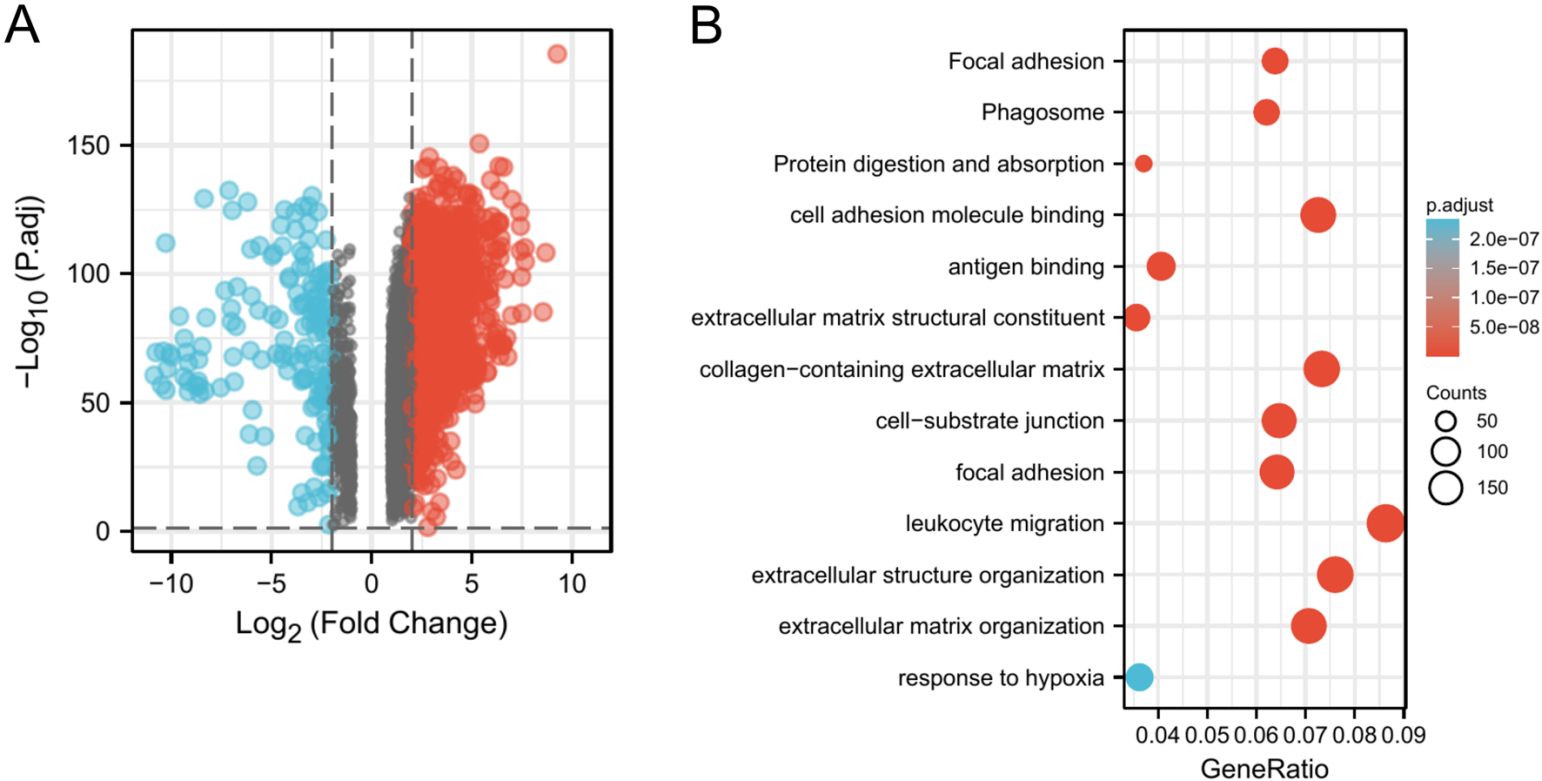
Gene differential expression and enrichment analysis from GEPIA2, (**A**) Pancreatic cancer differential gene from GEPIA2, up-regulated in red and down-regulated in blue, (**B**) GO/KEGG from the GEPIA2 pancreatic cancer differential gene.

A total of 9211 differentially expressed genes were screened, including 1279 up-regulated genes and 116 down-regulated genes (Fig. 1A).

We used GO/KEGG analysis of 2615 selected genes to identify hypoxia-related pathways. They identified hypoxia-related pathways: GO:0001666 (response to hypoxia, a total of 45 genes were identified) (Fig. 1B).

### Establishment of a prognostic model

**Figure 2:**
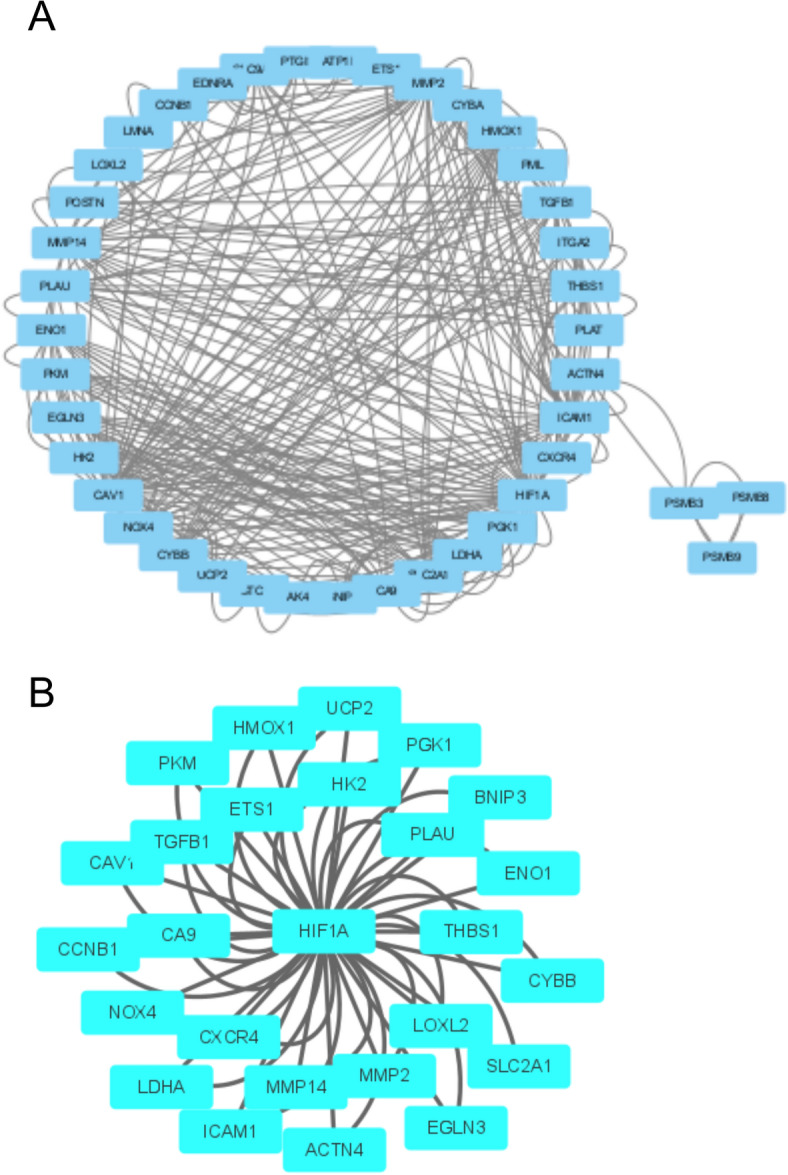
Visualization of genes associated with hypoxia pathways, (**A**) The PPI network in GO:0001666 and the (**B**) 25 genes in GO:0001666 are directly related to HIF1A.

**Figure 3:**
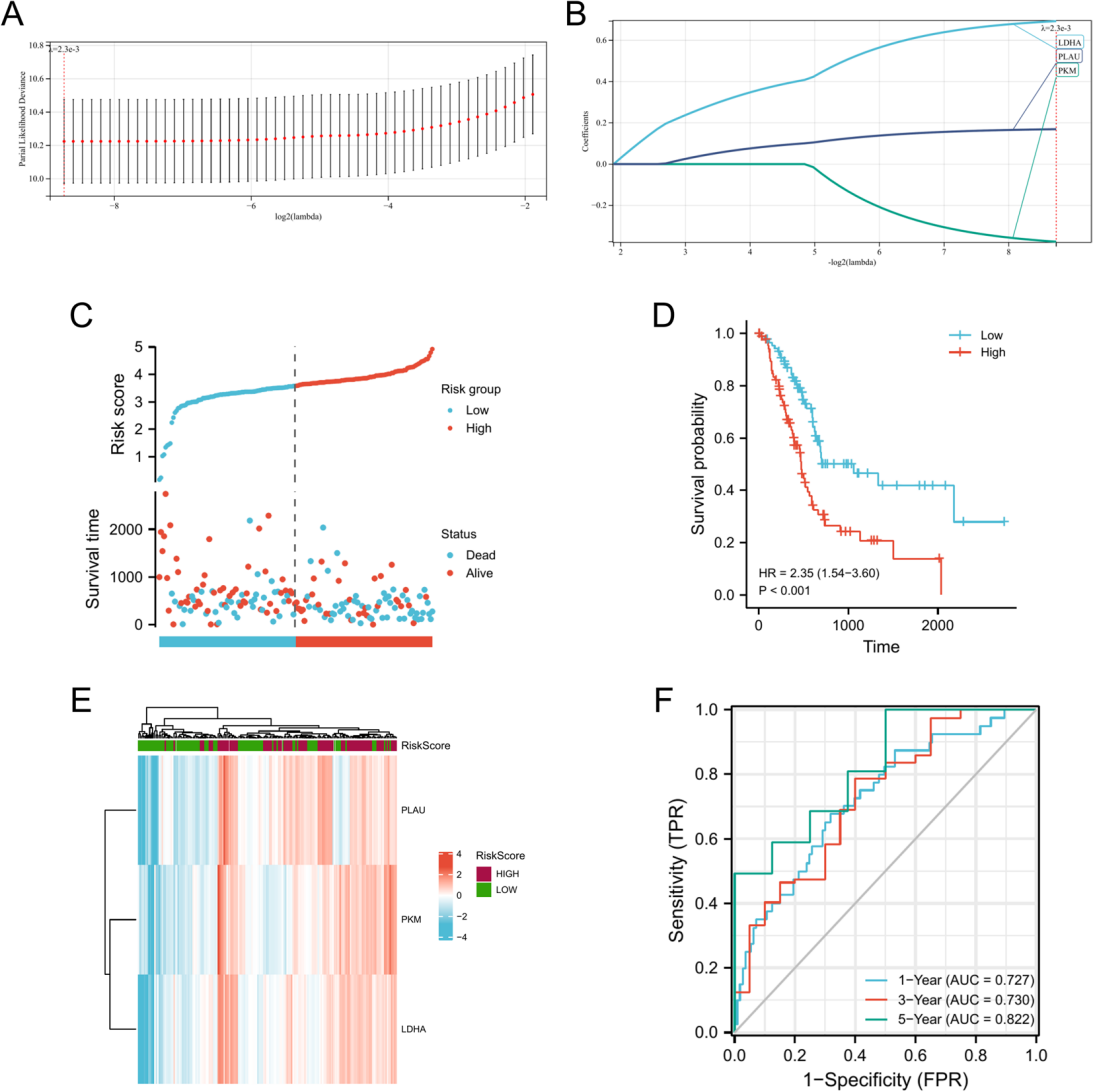
Risk score analysis, prognostic performance, and survival analysis of prognostic models, (**A**). The LASSO regression model of the 3 hypoxia-related genes performed by Lasso-ten-fold cross-validation, (**B**). The coefficient distribution in the LASSO regression model, (**C**). Risk scores and survival time distribution of hypoxia-related genes in the TCGA-PAAD cohort, (**D**). Kaplan–Meier analysis of OS survival between at-risk groups in the TCGA-PAAD cohort, (**E**). Heat map of gene expression of hypoxia-associated genes in the TCGA-PAAD cohort, (**F**). The ROC curves of the risk scoring model predict OS of 1-year, 3-year, and 5-year in the TCGA-PAAD cohort.

We screened out 25 genes directly related to hif1a through the STRING database (Fig. 2A,B). The key hypoxia genes with p < 0.05 were screened by univariate COX regression analysis (Supplementary file 1: Table S1). Next, according to the relationship between related genes and HIF1A in the string and the results of univariate COX regression, PKM, PLAU and LDHA were used for LASSO regression, and a prognostic model was established using LASSO regression (Fig. 3A,B).

We established a prognostic model formula for all cancer samples in the training set:

Risk Score = 0.693079351003975* expression level of LDHA − 0.376224999076138* expression level of PKM + 0.169271183333193* expression level of PLAU.

In order to verify if our model can accurately predict the prognosis of pancreatic cancer patients, 178 patients were divided into high-risk (n = 89) and low-risk (n = 89) groups (Fig. 3C). The log-rank test results suggested that the difference in survival time distribution between the risk score groups was statistically significant (Fig. 3D). The prognosis was worse in the group with a high-risk score. Similarly, we discovered that PKM, LDHA, and PLAU were mostly highly expressed in the high-risk group and mostly lowly expressed in the low-risk group (Fig. 3E). Time-dependent ROC analysis showed that the prognosis accuracy of 1-year OS was 0.727 (95% CI 0.596–0.792) and that of 3-year OS was 0.730 (95% CI 0.602–0.842). The 5-year prognostic accuracy of OS was 0.822 (95% CI 0.708–0.968) (Fig. 3F). These results indicate that the hypoxic-related gene prognosis model developed by our group can predict the prognosis of pancreatic cancer patients.

### Validation of prognostic models

**Figure 4:**
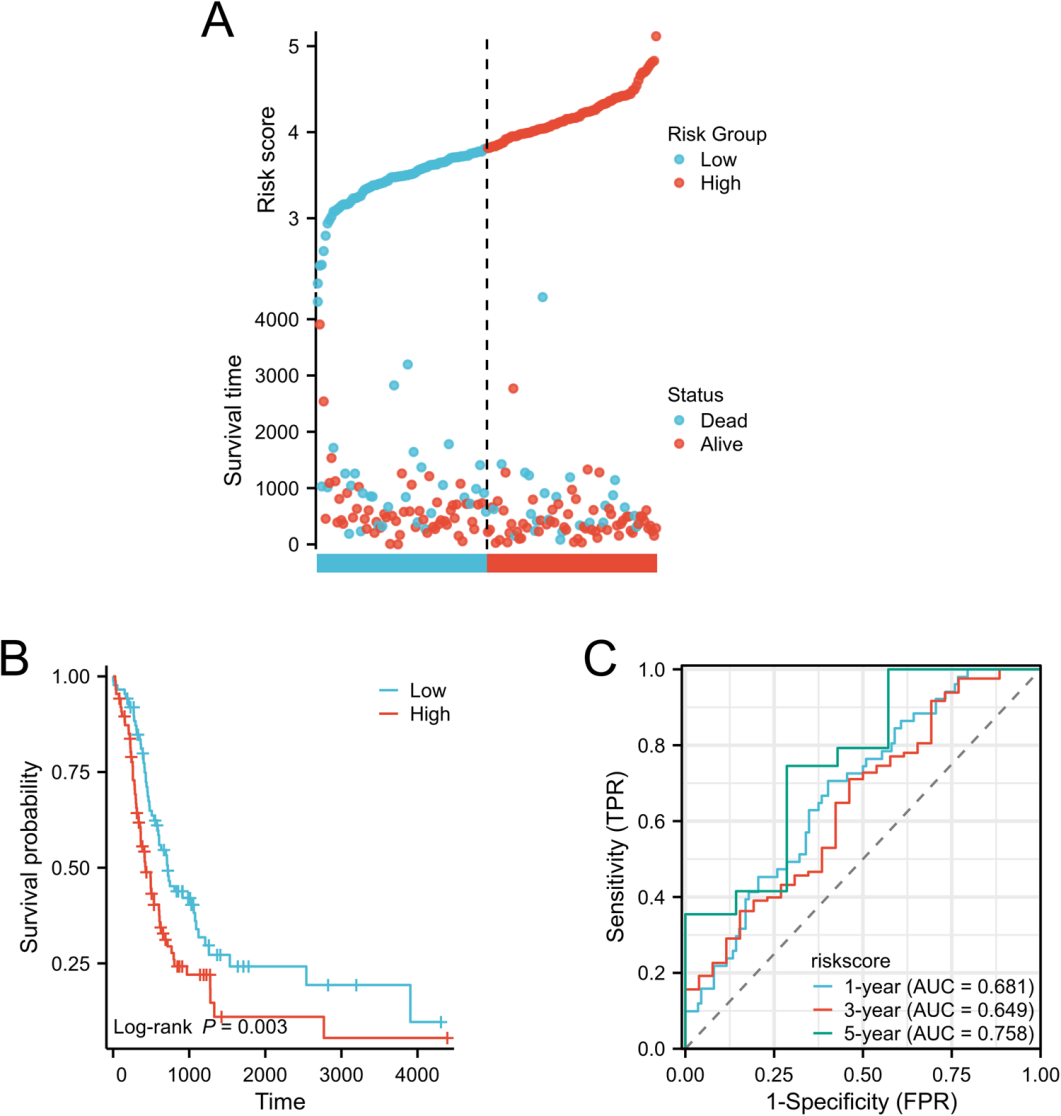
Analysis of the validation set prognostic model’s risk score, prognostic performance, and survival analysis, (**A**). The risk scores and survival time distribution of hypoxia-related genes comprise the verification set, (**B**). Kaplan–Meier survival analysis of OS between at-risk groups in the verification set, (**C**). The verification set ROC curves of the risk scoring model predicting 1-year, 3-year, and 5-year OS.

To verify the accuracy of the established prognostic model, we used GSE85916 and ICGC-PACA-AU as the verification set, collected data from 174 GSE85916 and ICGC-PACA-AU pancreatic cancer patients (79 were from GSE85916 and 95 from ICGC-PACA-AU), and calculated risk scores using the same formula as in the training set. We divided the verification set into a high-risk group (n = 87) and a low-risk group (n = 87) based on the median risk score (Fig. 4A). As with the training set, we observed shorter survival times in the peak risk group (Fig. 4B). Time-dependent ROC analysis revealed that 1-year OS prognostic accuracy was 0.681 (95% CI 0.629–0.863), and 3-year OS was 0.649 (95% CI 0.690–0.940). The 5-year prognostic accuracy of OS was 0.758 (95% CI 0.745–1.007) (Fig. 4C).

**Figure 5:**
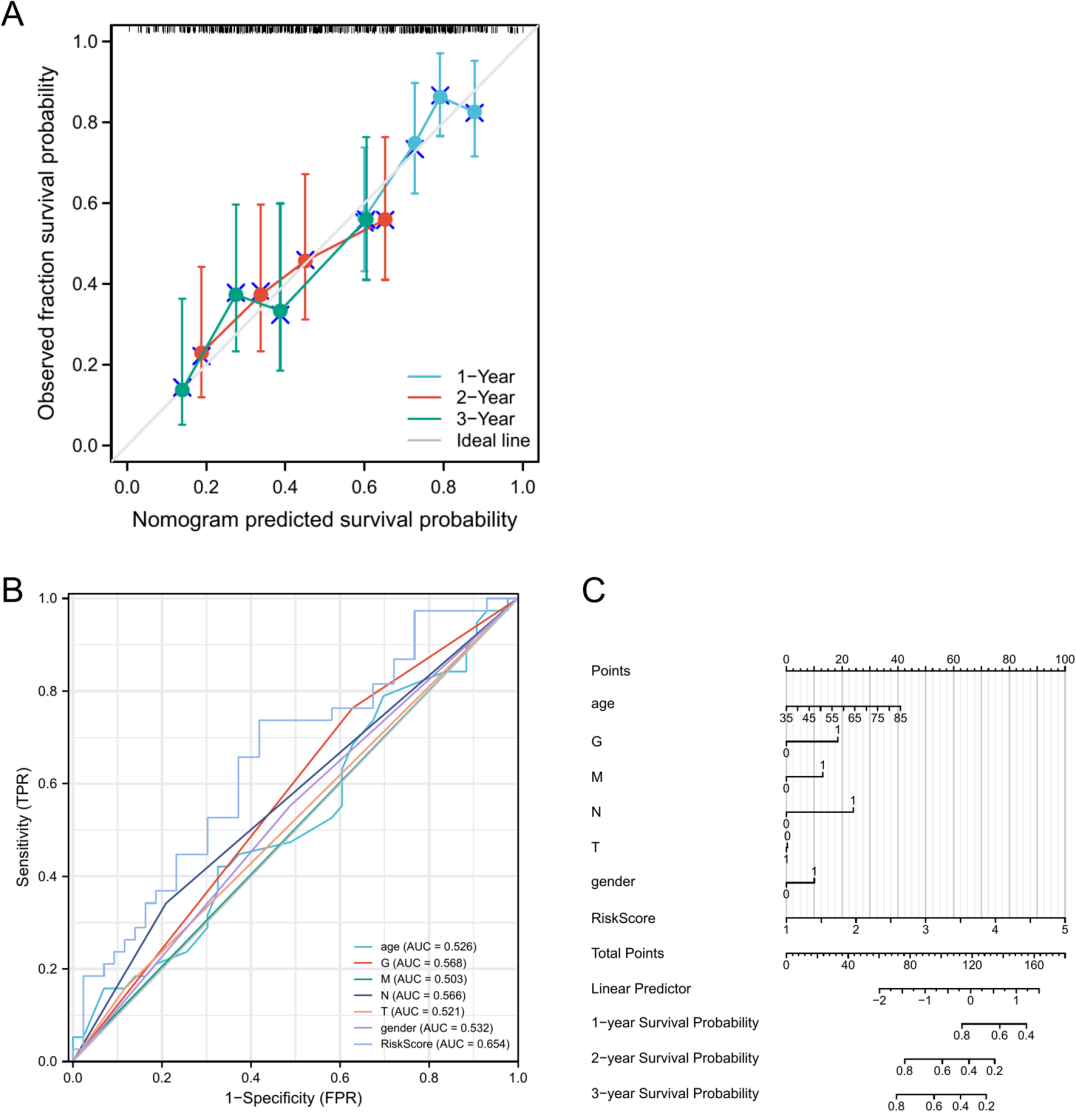
Prognosis of 1-year, 3-year, and 5-year OS in PAAD patients by nomogram, (**A**). nomogram calibration curves to predict 1-year, 3-year, and 5-year OS in TCGA-PAAD cohorts: (**B**) ROC curves for the prediction of survival by the risk score and other variables (age, gender, T stage, M stage, N stage, G stage); (**C**) nomogram of risk scores and traditional prognostic factors.

Moreover, we constructed a calibration curve, which showed that our model was in good agreement with the actual survival of PAAD patients (Fig. 5A). In addition to establishing a nomogram of risk scores and traditional prognostic factors (Fig. 5C), we discovered that our model had a higher AUC value than traditional clinical factors (Fig. 5B).

Furthermore, we conducted multivariate COX regression analyses on risk scores and clinical factors that may affect the prognosis of PAAD patients, such as T stage, gender, age, and histological grade, and the results indicated that the risk scores of our prognostic model could be used as independent risk factors in multivariate COX (Supplementary file 2: Table S2).

In conclusion, our model is capable of accurately predicting the prognosis of patients with pancreatic cancer.

### Correlation between risk scores and clinical features

**Figure 6:**
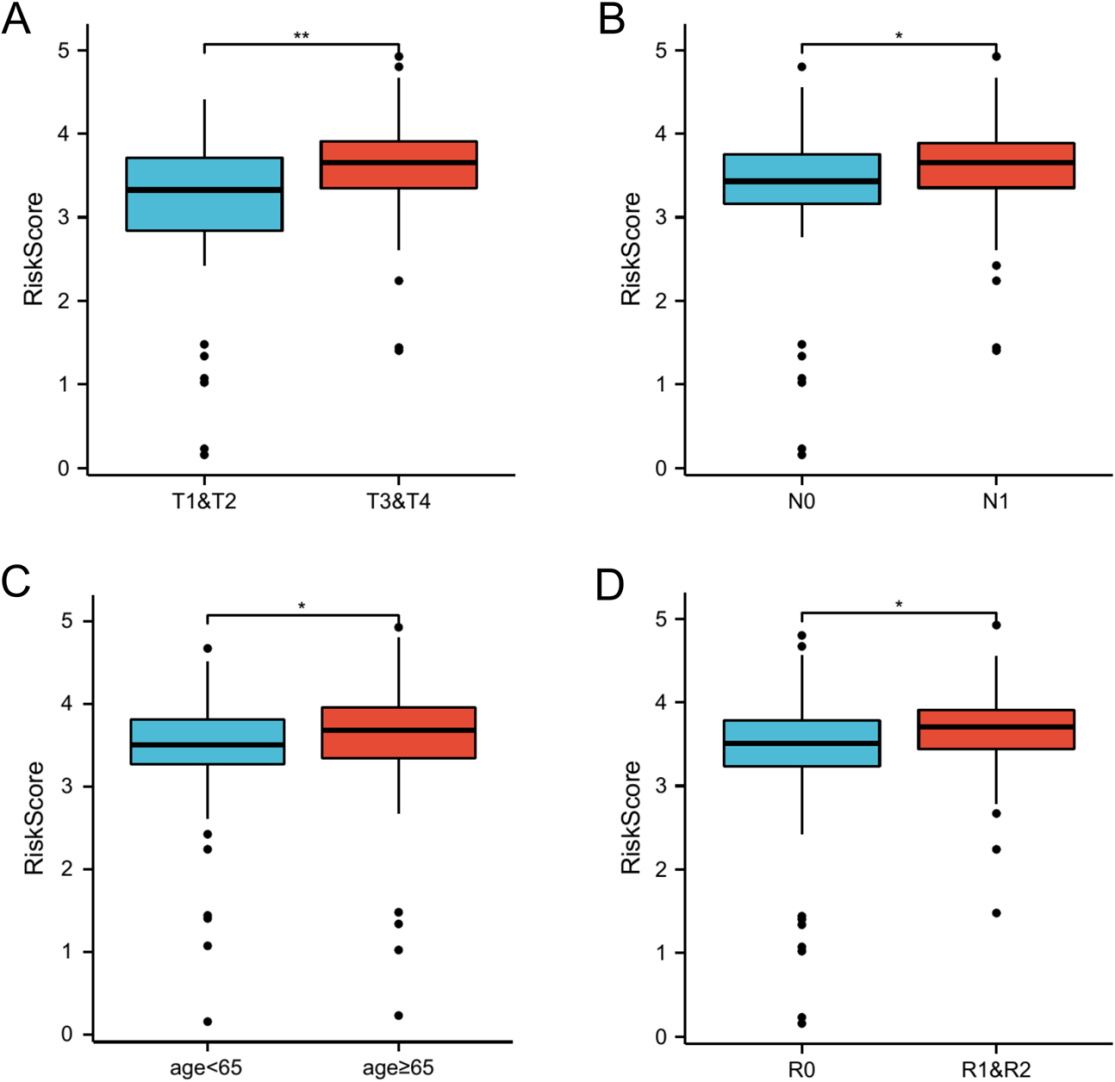
Association between clinical features and risk scores in the TCGA-PAAD dataset; correlation between clinical features and prognostic model risk scores in TCGA-PAAD cohort data: (**A**) T stage (T1 and T2 vs. T3 and T4), (**B**) N stage (N0 vs. N1); (**C**) AGE (age < 65 vs. age ≧65); (**D**) Residual tumor (R0 vs. R1 and R2).

The relationship between the risk score of our established prognostic model and the clinical features of TCGA-PAAD was also investigated. The distribution of risk scores varied significantly by T stage (Fig. 6A), N stage (Fig. 6B), age (Fig. 6C) and tumor residual (Fig. 6D).

### Immune cell infiltration level analysis

**Figure 7:**
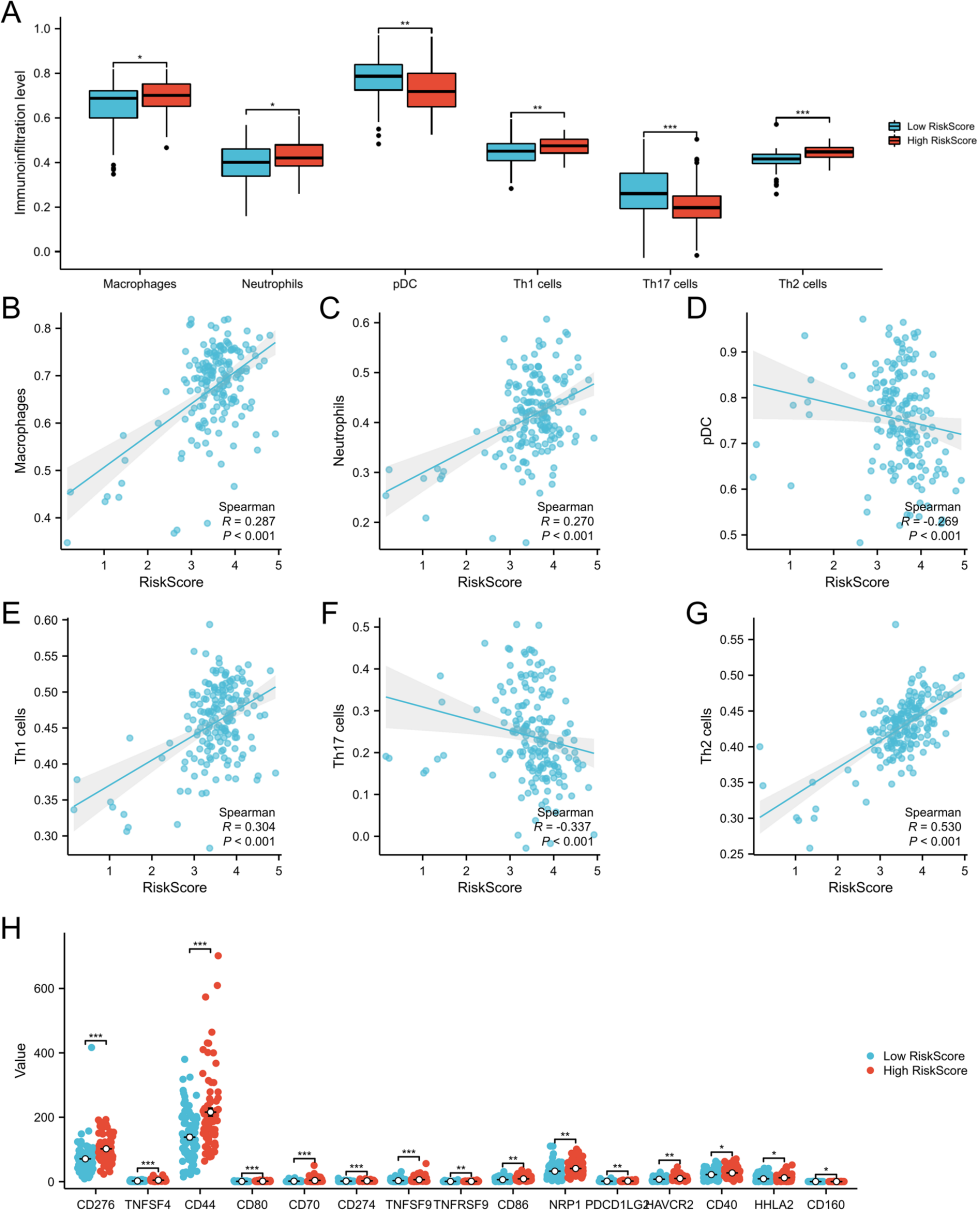
Immune infiltration and risk score association in the TCGA-PAAD dataset, (**A**). The box plot shows the level of immune cell infiltration between high-risk and low-risk groups, (**B**–**G**). Scatter plot of immune cell infiltration associated with risk score, (**H**) The box plot shows the level of immune checkpoint expression between high-risk and low-risk groups.

It was determined that neutrophils, Th1 cells, macrophages, and Th2 cells had a high degree of invasion, while PDC and Th17 cells had a low degree of invasion (Fig. 7A). Similarly, neutrophils, Th1 cells, macrophages, and Th2 cells were positively correlated with their risk scores, whereas PDC and Th17 cells were negatively correlated with their risk factors (Fig. 7B–G).

In our model, we also found that CD276, TNFSF4, CD44, CD80, CD70, CD274, TNFSF9, TNFRSF9, CD86, NRP1, PDCD1LG2, HAVCR2, CD40, HHLA2, and CD160 are differentially expressed between high-risk and low-risk patients. CD160 was highly expressed in the low-risk group, while the others were inversely expressed (Fig. 7H). Additionally, we identified that CD160 was negatively correlated with a risk score, whereas the others were positively correlated (Supplementary file 5: Fig. S1A–O).

### Real-time quantitative PCR

**Figure 8:**
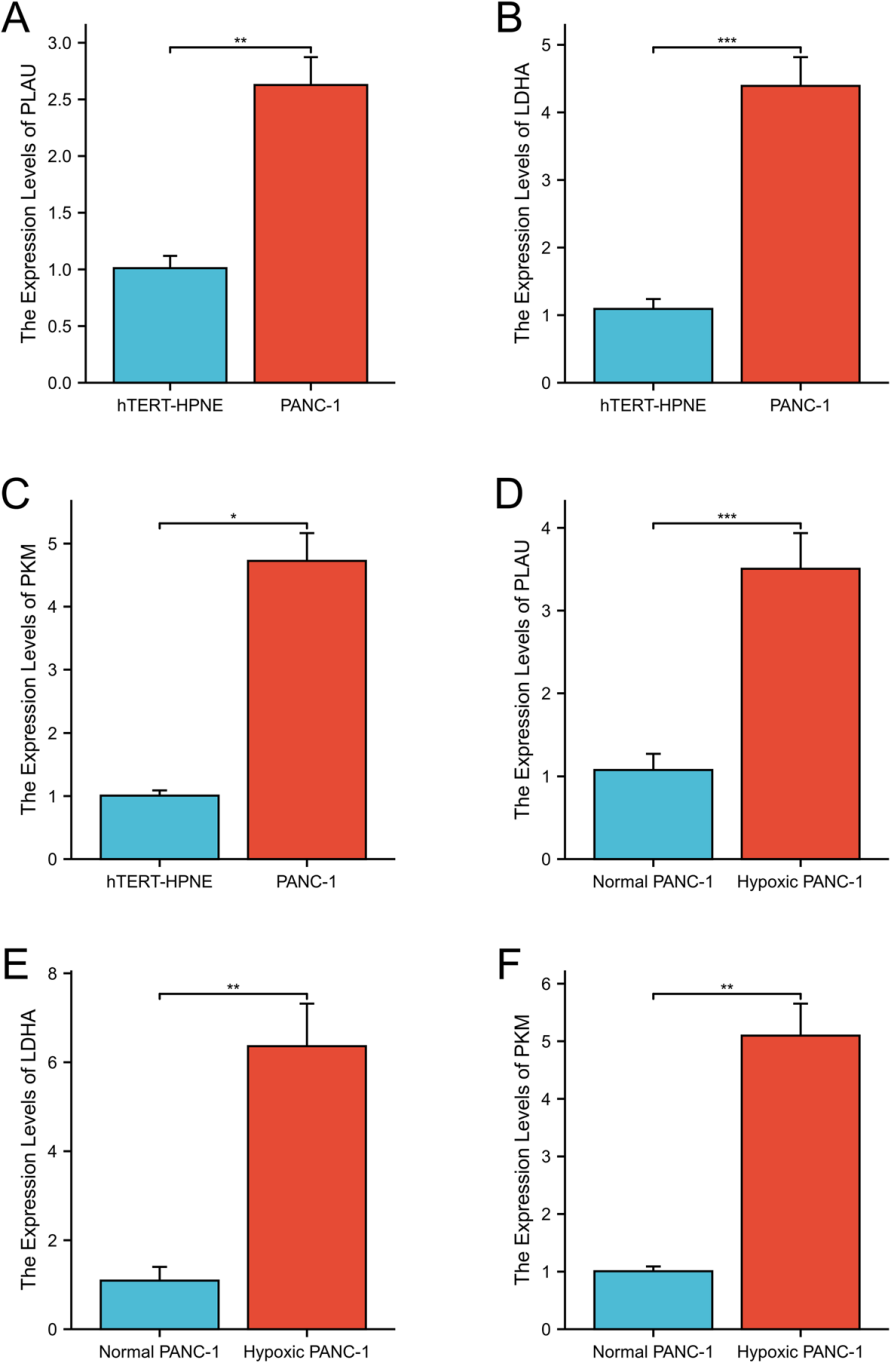
The gene was expressed in PANC-1 and hTERT-HPNE cells ((**A**) PLAU, (**B**) LDHA, and (**C**) PKM), and in hypoxic-treated PANC-1 cells and untreated PANC-1 cells ((**D**) PLAU, (**E**) LDHA, and (**F**) PKM).

According to the results of RT-PCR, we observed that PLAU, PKM, and LDHA were highly expressed in PANC-1 cells compared to hTERT-HPNE cells (Fig. 8A–C). The expressions of PKM and PLAU were lower in hypoxic-treated PANC-1 cell lines than in untreated cells, whereas the expressions of LDHA were higher in hypoxic-treated PANC-1 cell lines than in untreated cells (Fig. 8D–F).

## Discussion

Pancreatic cancer is one of the most deadly malignancies^18^. While survival rates for other major cancers have improved substantially, but pancreatic cancer survival rates have not improved^19^. Pancreatic cancer is usually detected at an advanced stage, and most treatment options are ineffective, resulting in a poor prognosis^5^. The need for accurate prognostic information is not only for patients but also for clinicians to choose active treatment interventions and anticipate significant clinical benefit. Therefore, a reliable prognostic model is required to predict the prognosis of pancreatic cancer patients.

Normal oxygen concentrations are necessary for the functioning and maintenance of aerobic organs^20^. Normal oxygen concentration is required for the normal functioning and maintenance of aerobic organs within an organization^21^. However, due to the uncontrolled growth and proliferation of tumor cells and abnormal tumor blood vessels, a large amount of nutrients and oxygen are consumed in tumor tissues, resulting in hypoxia^22^. In tumor tissues, the vascular network cannot form efficiently and promptly, and the neovascularization network has structural and functional abnormalities^23^. The aforementioned large number of neovascularization’s of non-functional or functionally impaired blood vessels is another significant cause of hypoxia^24^. The hypoxia induced factor (HIF) transcription factor family and its downstream related signaling pathways are primarily responsible for regulating the adaptive process that occurs in tumor cells under hypoxic conditions^25^.

With the development of research in recent years, the study of PAAD biomarkers, prognostic markers, and prognostic models has received a growing amount of attention. Through enrichment analysis of differential genes from GEPIA2, we identified hypoxia-related genes, and through the PPI network, we determined the gene directly related to HIF1A, which plays the most critical role in the process of tumor hypoxia. We selected hypoxia-related prognostic genes (PLAU, PKM, and LDHA) using single-factor Cox regression screening and established prognostic models using Lasso analysis. After validation and analysis, we discovered that our model can accurately predict the prognosis of patients with pancreatic cancer. The predictive efficacy of this model (area under the ROC curve) was greater than that of traditional clinical prognostic factors, according to our study. We also revealed that our prognostic model was strongly correlated with clinical features, with higher risk scores in T and N stages and tumor residual indicating relatively malignant stages and excessive tumor residual. This indicates that our model is helpful for clinicians to improve their prognostic judgment of pancreatic cancer patients in order to complete the treatment of pancreatic cancer more effectively and promptly and to provide a selection of prognostic interventions, which is anticipated to significantly improve patient survival and prognostic effects.

In addition to improving oxygen delivery by promoting angiogenesis and erythropoiesis, HIF-1 also adapts to anoxic environments by regulating metabolism to reduce the need for oxygen in cells.In glucose metabolism, HIF-1 converts oxidative metabolism to glycolysis by promoting the expression of glycolytic enzymes, reducing the need for oxygen^26^. PKM is one of the key enzymes in the conversion of glucose to pyruvate, and LDHA can catalyze the conversion of pyruvate to lactic acid^27,28^. These genes were found to contain sequences similar to HIF-1 binding sites in erythropoietin enhancers^29^. Under hypoxia conditions, HIF-1 can transform oxidative metabolism to glycolysis and enhance glycolysis by regulating the expression of the above genes, so as to reduce oxygen demand and maintain normal energy supply, while inhibiting the cell damage caused by hypoxia-induced reactive oxygen species generation^30^. PLAU encodes a secretory serine protease that converts plasminogen to plasminase^31^. PLAU has also been found to be involved in a variety of cancers and is associated with poor prognosis in a variety of cancers^32^; Some experimental studies have proved that PLAU overexpression is associated with poor prognosis of PAAD, and plays an important role in PAAD resistance, invasion and migration^33^. However, the role of PLAU in the anoxia process of pancreatic cancer has not been well understood, and our study aims to fill this gap.

Tumor progression and prognosis are closely related to immune cell infiltration in the tumor microenvironment^17^. We found that macrophages, neutrophils, Th1 cells, Th2 cells, and macrophages were more aggressive in the high-risk group, whereas PDC and Th17 cells were less aggressive. The tumor immune microenvironment in rapidly progressing PAAD patients is often associated with inadequate infiltration of immune cells^34^. Macrophages and neutrophils promote the suppression of immunosuppressive cells^35^. In our study, increased infiltration of these two types of cells in patients at high risk of hypoxia can increase the immunosuppressive tumor microenvironment and promote the progression of pancreatic cancer, which is associated with a poor prognosis. An immune checkpoint is a series of molecules expressed on immune cells that regulate immune activation^36^. Immune checkpoints play a crucial role in carcinogenesis by promoting the immunosuppressive effect of tumors^37^. Tumors can protect themselves from attack by stimulating immune checkpoint targets^38^. The immune checkpoints PD-L1, PD-L2, and CD276 were also upregulated in the hyperhypoxic risk group in our study. Our model began with hypoxic-related genes in pancreatic cancer and simultaneously examined the relationship between the model and the immune microenvironment in order to examine the associated immune infiltration in high and low risk patient groups. We determined that our hypoxic-related gene model is highly correlated with the immune microenvironment, providing a novel method for predicting the prognosis of pancreatic cancer based on the tumor microenvironment. Immune checkpoints are a series of molecules expressed on immune cells that regulate the degree of immune activation and play an important role in preventing autoimmunity (when the immune function goes wrong and attacks healthy cells).

In addition, we verified the expression difference of the three genes in pancreatic cancer cells and normal pancreatic ductal epithelial cells by RT-qPCR experiments, and also confirmed that the expression of the three genes differed in pancreatic cancer cells before and after hypoxia, indicating that these three genes have the potential to become hypoxia-related prognostic markers of pancreatic cancer.

Our model has the potential to be a prognostic model related to the tumor microenvironment of pancreatic cancer and can reliably predict the prognosis of patients based on the impact of the tumor microenvironment on pancreatic cancer.

## Methods and materials

### Cell culture and reagents

Human pancreatic cancer cell PANC-1 were purchased from PROCELL and human normal pancreatic ductal cell hTERT-HPNE were purchased from CELL RESEARCH. Both kinds of cells were stored in the sample bank of the Affiliated Hospital of Qingdao University by liquid nitrogen.Cells were cultured in DMEM high glucose medium supplemented with 10% fetal bovine serum and 1% penicillin/streptomycin (purchased by Meilunbio) in a wet incubator with 5% carbon dioxide at 37 °C. Hypoxia-treated PANC-1 cells were cultured for 24 h in an anoxic incubator (Ruskinn Invivo2 400 Hypoxia workstation).

### Search for genes in hypoxia

We used the online database GEPIA2^39^ to identify the differentially expressed genes of pancreatic cancer and took log2FoldChange (logFC) and P-values as the screening conditions for differentially expressed genes. We filter for P-value < 0.05 and | log2FC |> 1 gene as a difference between 2 conditions: log2FC > 2.5 for up-regulated, log2FC < − 2.5 for down-regulated. Afterwards, volcano maps were used to visualize these differential genes.

Gene ontology (GO) and the Kyoto Encyclopedia of Genes and Genomes (KEGG) can enrich and annotate genes and find related pathways. Genomes were enriched and analyzed using R. Main package clusterProfiler [version 3.14.3]; org.Hs.eg.db package [version 3.10.0]; and GOplot package [version 1.0.2]. To identify the hypoxia-related enrichment pathways and the enriched genes.

### Identification of prognostic genes

Our training set consisted of 178 PAAD samples from the TCGA databases (Supplementary file 3: Table S3).

Previous research indicated that HIF1A plays a crucial role in hypoxia^40^. We mapped the PPI network of hypoxia-related genes by string, and looked for genes directly associated with HIF1A as key genes.

Single-factor COX regression was performed for the above key genes using the "survival" package. In p < 0.05 genes, the two genes with the lowest p value and the gene with the closest relationship with HIF1A (with the largest combined score) were selected as the genes to establish the prognostic model. The prognostic model was established using LASSO regression^41^.

### Construction of a prognostic model

Risk scores were calculated based on standardized PAAD mRNA expression data in TCGA.

In this study, the R software package glmnet was used to integrate survival time, survival state, and gene expression data, and regression analysis was performed using the lasso-cox method. In addition, we also implemented a tenfold cross-validation procedure to determine the optimal model. PAAD patients were divided into high-risk and low-risk groups based on the median risk score for OS survival analysis. An ROC curve was developed using the "timeROC" package to evaluate the prognostic effect of the model.

### Validation of prognostic models

To verify the accuracy of the established prognostic model, we used GSE85916 and ICGC-PACA-AU as the verification set(Supplementary file 4: Table S4).

Meanwhile, we use the "rms" and "survival" packages to evaluate the effect of the model’s prediction on the actual outcome by plotting the actual probability and the probability predicted by the model in different situations in the graph.

The "pROC" package was then used to plot ROC curves for risk score and associated clinical variables in order to determine whether our model has higher predictive power for prognosis compared with traditional clinical prognosis scoring systems.

Multivariate Cox regression analyses were performed for clinicopathologic parameters such as histological grade and T stage to evaluate whether the risk scoring system could be used as an independent predictor. We also analyzed the relationship between risk score and the clinical characteristics of TCGA-PAAD cohort patients.

### Immune cell infiltration level analysis

On the basis of the ssGSEA algorithm provided by the R-package GSVA, the immune infiltration corresponding to the prognostic model was calculated using 24 types of immune cell markers from the Immunity article^21^.

Concurrently, the expression of immune checkpoint related molecules in patients with pancreatic cancer was determined.

### Real-time quantitative PCR

Total RNA was extracted from PANC-1, hTERT-HPNE, and PANC-1 cultured for 24 h after hypoxia using the anaerobic incubator. Reversely transcribed was performed using the PrimeScrip RT-PCR kit (Takara, Japan), and RT-qPCR was performed on a Roche instrument with SYBR PreMix Ex Taq (Takara, Japan). Primer sequences used in this study are shown as follows: PLAU forward: GGGAATGGTCACTTTTACCGAG, PLAU reverse: GGGCATGGTACGTTTGCTG; GAPDH Forward: GGAGCGAGATCCCTCCAAAAT, GAPDH Reverse: GGCTGTTGTCATACTTCTCATGG; PKM Forward CTGAAGGCAGTGATGTGGCC, PKM Reverse ACCCGGAGGTCCACGTCCTC; LDHA Forward GGCCTGTGCCATCAGTA: LDHA Reverse CAAGCCACGTAGGTCAA.

### Statistical analysis

SPSS 24.0 software (IBM Corp., NY, USA) and GraphPad Prism 8 (GraphPad, USA) were used for statistical analysis. The Student t-test was used to analyze the difference in genes. |logFC|> 1 and P < 0.05 were set as thresholds to choose the significance of the differential expression gene. Univariate and multivariate COX analysis, the log-rank test, and logistic regression analysis were employed.

## Supplementary Information

Supplementary Table S1.

Supplementary Table S2.

Supplementary Table S3.

Supplementary Table S4.

Supplementary Figure S1.

## Data Availability

The datasets used and/or analyzed during this study are available upon reasonable request from the corresponding author.
