# Supplementary material for: Effects of anoxic prognostic model on immune microenvironment in pancreatic cancer
Source: Sci Rep. 2023 Jun 5;13:9104. doi: 10.1038/s41598-023-36413-9 (PMC10241784; doi:10.1038/s41598-023-36413-9)
Supplement: Supplementary file 1 — Supplementary Table S1. [file 41598_2023_36413_MOESM1_ESM.pdf]

Supplementary file 1： TABLE S1: Univariate COX regression of hypoxia gene

Characteristics	Total(N)	Univariate analysis		Multivariate analysis	
		Hazard ratio (95% CI)	P value	Hazard ratio (95% CI)	P value
SLC2A1	178	1.001 (1.000-1.002)	0.051	0.998 (0.996-1.000)	**0.046**
LDHA	178	1.001 (1.001-1.002)	**<0.001**	1.003 (1.001-1.004)	**<0.001**
PTGS2	178	1.008 (1.002-1.015)	**0.011**	1.006 (0.997-1.015)	0.196
VCAM1	178	1.009 (1.002-1.016)	**0.013**	1.006 (0.997-1.015)	0.205
PGF	178	1.000 (0.976-1.024)	0.990		
UBC	178	1.000 (1.000-1.001)	0.307		
MMP14	178	1.001 (1.000-1.002)	**0.030**	0.996 (0.993-0.999)	**0.022**
PGK1	178	1.001 (1.000-1.002)	**0.024**	0.999 (0.996-1.002)	0.397
PKM	178	1.000 (1.000-1.001)	**0.034**	1.000 (0.999-1.001)	0.550
ENO1	178	1.001 (1.000-1.001)	**0.001**	1.000 (0.999-1.001)	0.767
LOXL2	178	1.005 (1.001-1.009)	**0.014**	1.010 (0.997-1.022)	0.134
HK2	178	1.006 (1.000-1.012)	0.067	1.003 (0.988-1.018)	0.725
CA9	178	1.000 (1.000-1.001)	0.084	1.000 (1.000-1.001)	0.479
CYBB	178	1.001 (0.988-1.014)	0.901		
TGFB1	178	1.001 (0.993-1.010)	0.749		
UCP2	178	0.996 (0.991-1.000)	0.068	0.996 (0.991-1.002)	0.184
BNIP3	178	0.988 (0.971-1.004)	0.139		
RBX1	178	1.009 (0.997-1.021)	0.125		
CAV1	178	1.005 (1.002-1.008)	**0.002**	1.001 (0.995-1.007)	0.850
THBS1	178	1.001 (1.000-1.003)	0.053	1.002 (0.999-1.004)	0.165
BNIP3L	178	1.010 (1.001-1.019)	**0.038**	0.983 (0.962-1.005)	0.134
EGLN3	178	0.999 (0.997-1.002)	0.614		
RHOA	178	1.002 (1.000-1.004)	0.056	1.000 (0.998-1.003)	0.800
ETS1	178	1.003 (0.996-1.010)	0.344		
HMOX1	178	1.002 (0.992-1.011)	0.742		

Characteristics	Total(N)	Univariate analysis		Multivariate analysis	
		Hazard ratio (95% CI)	P value	Hazard ratio (95% CI)	P value
ICAM1	178	1.002 (0.995-1.008)	0.597		
VEGFC	178	0.980 (0.944-1.017)	0.290		
PLAU	178	1.002 (1.001-1.004)	**<0.001**	1.003 (1.001-1.006)	**0.011**
CXCR4	178	0.999 (0.997-1.001)	0.399		
TWIST1	178	1.003 (0.988-1.018)	0.737		
CCNB1	178	1.022 (1.009-1.034)	**<0.001**	1.014 (0.994-1.034)	0.185
NOX4	178	1.025 (0.999-1.052)	0.061	0.992 (0.946-1.040)	0.728
MMP2	178	1.000 (1.000-1.001)	0.065	1.000 (0.998-1.001)	0.668
