# Supplementary material for: Effects of anoxic prognostic model on immune microenvironment in pancreatic cancer
Source: Sci Rep. 2023 Jun 5;13:9104. doi: 10.1038/s41598-023-36413-9 (PMC10241784; doi:10.1038/s41598-023-36413-9)
Supplement: Supplementary file 2 — Supplementary Table S2. [file 41598_2023_36413_MOESM2_ESM.pdf]

Supplementary file 2: TABLE S2: Multivariate COX regression results of clinical factors and Riskscore

Characteristics	HR Multivariate analysis	CI Low Multivariate analysis	CI High Multivariate analysis	P value Multivariate analysis
age	1.02823113518446	0.996820572089267	1.06063146865718	0.0786141396444288
G				
1	Reference			
0	0.533614805400837	0.269231469713319	1.05762064459319	0.0719457881057535
M				
0	Reference			
1	1.56028887074471	0.348161862192473	6.99244123075134	0.561036005348444
N				
1	Reference			
0	0.44293827296163	0.191133744134995	1.02647658864288	0.0575614200467622
T				
1	Reference			
0	1.01391010790564	0.337801200342144	3.04325060382259	0.980346965719799
gender				
1	Reference			
0	0.711544359190197	0.333169619748989	1.5196324787261	0.379374726874095
Riskscore	2.3370576015516	1.13687494738051	4.80425595229697	0.0209503712175753
