# Supplementary material for: Effects of anoxic prognostic model on immune microenvironment in pancreatic cancer
Source: Sci Rep. 2023 Jun 5;13:9104. doi: 10.1038/s41598-023-36413-9 (PMC10241784; doi:10.1038/s41598-023-36413-9)
Supplement: Supplementary file 3 — Supplementary Table S3. [file 41598_2023_36413_MOESM3_ESM.pdf]

Supplementary file 3：TABLE S3: training set consisted of 178 PAAD samples are from the TCGA databases

Sample id	LDHA	PKM	PLAU
TCGA-2J-AAB1-01A-11R-A41B-07	8.285744503	8.647107564	5.946855848
TCGA-2J-AAB4-01A-12R-A41B-07	9.072830326	9.278884281	8.022936252
TCGA-2J-AAB6-01A-11R-A41B-07	8.744353104	9.779004767	11.12024616
TCGA-2J-AAB8-01A-12R-A41B-07	8.219023427	9.38277134	8.733800729
TCGA-2J-AAB9-01A-11R-A41B-07	7.630354985	8.647635963	7.784571466
TCGA-2J-AABA-01A-21R-A41B-07	8.536648688	9.454958708	6.946978598
TCGA-2J-AABE-01A-12R-A41B-07	7.77834546	9.123625907	7.98948772
TCGA-2J-AABF-01A-31R-A41B-07	7.696771693	9.05064183	6.821116357
TCGA-2J-AABH-01A-21R-A41B-07	8.294322108	8.346418344	6.98615445
TCGA-2J-AABI-01A-12R-A41B-07	8.697382467	9.598148379	8.9081938
TCGA-2J-AABK-01A-31R-A41B-07	7.878117678	8.751430587	5.304930253
TCGA-2J-AABO-01A-21R-A41B-07	8.59105185	10.10261657	8.446882194
TCGA-2J-AABP-01A-11R-A41B-07	8.21610727	9.739662665	7.886650353
TCGA-2J-AABR-01A-11R-A41B-07	8.12610159	8.760675364	6.749921574
TCGA-2J-AABT-01A-11R-A41B-07	7.897938766	8.117667077	5.934240647
TCGA-2J-AABU-01A-11R-A41B-07	8.833830268	10.10876008	8.509767713
TCGA-2J-AABV-01A-12R-A41B-07	5.55283716	5.704590747	3.175915163
TCGA-2L-AAQA-01A-21R-A38C-07	8.95562543	9.452983862	7.819220848
TCGA-2L-AAQE-01A-11R-A39D-07	8.255557299	9.817545495	8.67905844
TCGA-2L-AAQI-01A-12R-A39D-07	9.120963915	9.74207144	6.799994836
TCGA-2L-AAQJ-01A-12R-A39D-07	8.50826253	8.922998024	7.746738153
TCGA-2L-AAQL-01A-11R-A38C-07	7.28758019	8.818512052	7.319948055
TCGA-2L-AAQM-01A-11R-A39D-07	8.180494478	9.088806763	2.478003045
TCGA-3A-A9I5-01A-11R-A38C-07	7.979627259	9.035336033	6.101539819
TCGA-3A-A9I7-01A-21R-A38C-07	8.24141801	8.86646415	7.85950222
TCGA-3A-A9I9-01A-11R-A38C-07	7.422895129	7.760800299	6.87834029
TCGA-3A-A9IB-01A-21R-A39D-07	9.501008563	9.43216866	9.643636423
TCGA-3A-A9IC-01A-11R-A38C-07	8.682873503	9.264450139	8.983899437
TCGA-3A-A9IH-01A-12R-A39D-07	9.315421887	10.15558827	7.874484303
TCGA-3A-A9IJ-01A-11R-A39D-07	5.419365378	8.317803901	2.637784871
TCGA-3A-A9IL-01A-11R-A38C-07	5.220693153	8.288814822	4.951152098
TCGA-3A-A9IN-01A-11R-A39D-07	5.31342033	7.718888896	4.136369404
TCGA-3A-A9IO-01A-11R-A38C-07	4.246956412	8.698965902	3.306327399
TCGA-3A-A9IR-01A-11R-A38C-07	5.060242589	8.104930463	3.336269249
TCGA-3A-A9IS-01A-21R-A39D-07	3.670426561	7.675039394	2.95854672
TCGA-3A-A9IU-01A-11R-A39D-07	8.898647902	9.677019544	8.820797938
TCGA-3A-A9IV-01A-11R-A41B-07	7.062994335	8.098857912	5.411596206
TCGA-3A-A9IX-01A-11R-A41B-07	7.445995532	8.294416955	6.461029902
TCGA-3A-A9IZ-01A-12R-A41B-07	8.863693258	10.04854301	7.926448251
TCGA-3A-A9J0-01A-11R-A41B-07	8.597446351	10.63480027	8.030082584

TCGA-3E-AAAY-01A-11R-A38C-07	8.075284823	8.856913296	7.154134315
TCGA-3E-AAAZ-01A-11R-A38C-07	7.848727281	8.754490424	7.210639099
TCGA-F2-6879-01A-11R-2156-07	8.861326758	8.853874845	5.740338151
TCGA-F2-6880-01A-11R-2156-07	4.166808905	5.018351807	2.603224886
TCGA-F2-7273-01A-11R-2156-07	8.191293823	8.172360624	6.191749278
TCGA-F2-7276-01A-11R-2156-07	7.913455167	8.400897898	7.387716006
TCGA-F2-A44G-01A-11R-A26U-07	9.213070328	9.436818058	8.398521212
TCGA-F2-A44H-01A-11R-A26U-07	6.837034884	8.341674694	8.036206019
TCGA-F2-A7TX-01A-33R-A38C-07	8.560330118	9.98547244	9.207266891
TCGA-F2-A8YN-01A-11R-A37L-07	7.978270624	8.559099589	7.91105667
TCGA-FB-A4P5-01A-11R-A26U-07	8.115074668	8.691778099	6.838051177
TCGA-FB-A4P6-01A-12R-A26U-07	7.832236669	8.442020062	6.034402822
TCGA-FB-A545-01A-11R-A26U-07	9.600098504	9.667388436	8.833073587
TCGA-FB-A5VM-01A-11R-A32O-07	8.33549136	9.446505122	9.412371705
TCGA-FB-A78T-01A-12R-A32O-07	8.282854338	9.282529782	6.625541306
TCGA-FB-A7DR-01A-21R-A33R-07	8.234938441	8.336613464	9.001020335
TCGA-FB-AAPP-01A-12R-A41B-07	7.060976214	7.449427397	3.021938545
TCGA-FB-AAPQ-01A-11R-A41B-07	9.235174514	9.917417675	6.263640825
TCGA-FB-AAPS-01A-12R-A39D-07	7.804928292	8.609325802	9.212509626
TCGA-FB-AAPU-01A-31R-A41B-07	9.018848944	9.444719488	5.816650712
TCGA-FB-AAPY-01A-11R-A41B-07	8.098333294	8.9906019	6.926385205
TCGA-FB-AAPZ-01A-11R-A41B-07	8.143461586	8.843205286	7.18807596
TCGA-FB-AAQ0-01A-31R-A41B-07	9.109247098	9.247755694	6.67179229
TCGA-FB-AAQ1-01A-12R-A41B-07	9.133569259	9.547364765	8.19149053
TCGA-FB-AAQ2-01A-31R-A41B-07	9.02493502	9.699359625	8.263296843
TCGA-FB-AAQ3-01A-11R-A41B-07	8.172224862	8.052814213	7.144982455
TCGA-FB-AAQ6-01A-11R-A41B-07	8.39568897	9.549606758	6.430133659
TCGA-H6-8124-01A-11R-2404-07	9.707051803	11.06190009	8.220548861
TCGA-H6-A45N-01A-11R-A26U-07	7.655474012	8.805965094	7.499178588
TCGA-H8-A6C1-01A-11R-A32O-07	7.464161611	8.125938887	6.389615698
TCGA-HV-A5A3-01A-11R-A26U-07	8.904129825	8.925418324	7.90736175
TCGA-HV-A5A4-01A-11R-A26U-07	8.242132667	9.003279942	8.273924576
TCGA-HV-A5A5-01A-11R-A26U-07	7.733084027	9.182751171	7.37366511
TCGA-HV-A5A6-01A-11R-A26U-07	8.254860543	8.991429363	8.082782215
TCGA-HV-A7OL-01A-11R-A33R-07	8.355877807	9.573668583	7.586744571
TCGA-HV-A7OP-01A-11R-A33R-07	5.625806417	7.937748087	2.905024262
TCGA-HV-AA8V-01A-11R-A41B-07	8.093360537	9.029354161	9.110378403
TCGA-HV-AA8X-01A-11R-A39D-07	8.397830144	9.331016148	6.295375482
TCGA-HZ-7289-01A-11R-2156-07	8.718837068	7.690135589	4.898899671
TCGA-HZ-7918-01A-11R-2156-07	8.09803601	8.200897485	6.79067634
TCGA-HZ-7919-01A-11R-2156-07	9.211646492	9.889435372	8.006441157
TCGA-HZ-7920-01A-11R-2204-07	8.405396484	8.603213606	6.786030473
TCGA-HZ-7922-01A-11R-2156-07	8.999938489	9.927445671	8.346789482
TCGA-HZ-7923-01A-12R-2156-07	8.08154469	8.45802138	7.22184754

TCGA-HZ-7924-01A-11R-2156-07	7.918692555	8.356730434	4.656820668
TCGA-HZ-7925-01A-11R-2156-07	8.866150249	9.127247197	8.387626242
TCGA-HZ-7926-01A-11R-2156-07	8.737584925	8.730906489	8.603096692
TCGA-HZ-8001-01A-11R-2204-07	8.183038563	9.202389637	7.98073053
TCGA-HZ-8002-01A-11R-2204-07	8.200461298	8.717785365	7.52167036
TCGA-HZ-8003-01A-21R-2204-07	6.506433055	7.098127945	6.806431554
TCGA-HZ-8005-01A-11R-2204-07	9.922245212	10.65887738	9.449670519
TCGA-HZ-8315-01A-11R-2404-07	8.326279527	8.857099032	8.966984053
TCGA-HZ-8317-01A-11R-2404-07	7.772880022	7.792999954	7.072771947
TCGA-HZ-8519-01A-11R-2404-07	7.205759305	8.204694781	5.500262436
TCGA-HZ-8636-01A-21R-2404-07	8.826087786	9.404812433	8.334930249
TCGA-HZ-8637-01A-11R-2404-07	7.140291435	8.169861145	6.620724183
TCGA-HZ-8638-01A-11R-2404-07	8.132395665	8.543337865	5.336099129
TCGA-HZ-A49G-01A-11R-A26U-07	7.973790734	8.814019067	7.976285937
TCGA-HZ-A49H-01A-11R-A26U-07	7.611099063	7.694291991	5.746038239
TCGA-HZ-A49I-01A-12R-A26U-07	8.400717192	9.834021045	7.801847289
TCGA-HZ-A4BH-01A-11R-A26U-07	8.915998794	9.743348012	7.956341512
TCGA-HZ-A4BK-01A-11R-A26U-07	7.729921278	8.874927663	7.957964688
TCGA-HZ-A77O-01A-11R-A33R-07	8.624531802	9.375191272	7.450160111
TCGA-HZ-A77P-01A-11R-A33R-07	8.253344721	8.258756683	5.927725318
TCGA-HZ-A77Q-01A-11R-A36G-07	8.856450724	9.026388038	9.061422987
TCGA-HZ-A8P1-01A-11R-A37L-07	7.803359268	8.589105864	5.953110559
TCGA-HZ-A9TJ-01A-11R-A41I-07	7.666078602	8.48147399	6.343621134
TCGA-HZ-A9TJ-06A-11R-A41B-07	8.116881444	8.919976863	6.063355677
TCGA-IB-7644-01A-11R-2156-07	8.469251556	8.528362164	6.428749987
TCGA-IB-7645-01A-22R-2204-07	8.354493518	9.051870553	8.082972082
TCGA-IB-7646-01A-11R-2156-07	9.26015819	9.445851881	8.575439808
TCGA-IB-7649-01A-11R-2156-07	8.438770787	8.378237467	6.717248963
TCGA-IB-7651-01A-11R-2156-07	8.996838886	9.425577742	6.755602156
TCGA-IB-7652-01A-11R-2156-07	8.051862546	8.892225163	7.187365543
TCGA-IB-7654-01A-11R-2156-07	7.48128707	8.1828376	7.099533787
TCGA-IB-7885-01A-11R-2156-07	8.719581023	9.38082408	9.079913763
TCGA-IB-7886-01A-11R-2156-07	8.661440101	9.477417225	8.665099978
TCGA-IB-7887-01A-11R-2156-07	9.372842753	9.467657714	8.547801248
TCGA-IB-7888-01A-11R-2156-07	8.446235873	8.761190166	6.507810785
TCGA-IB-7889-01A-11R-2156-07	7.973942716	9.153916611	7.585968683
TCGA-IB-7890-01A-12R-2204-07	9.441239356	9.623679161	8.797873104
TCGA-IB-7891-01A-11R-2204-07	7.960496892	8.819211311	7.588500821
TCGA-IB-7893-01A-11R-2204-07	9.726746488	9.724949914	10.14302294
TCGA-IB-7897-01A-21R-2204-07	7.932120267	8.466871439	7.137572643
TCGA-IB-8126-01A-11R-2404-07	6.226974312	6.162312077	5.659526118
TCGA-IB-8127-01A-11R-2404-07	8.977340744	9.23812323	8.034966581
TCGA-IB-A5SO-01A-11R-A32O-07	7.757736423	9.136980111	7.92260515
TCGA-IB-A5SP-01A-11R-A32O-07	7.736757667	8.983531102	5.354695068

TCGA-IB-A5SQ-01A-11R-A320-07	8.098518687	9.259864116	9.529284637
TCGA-IB-A5SS-01A-11R-A320-07	9.775967301	10.1985559	9.591076797
TCGA-IB-A5ST-01A-11R-A320-07	8.050992491	8.42137031	7.449841322
TCGA-IB-A6UF-01A-23R-A33R-07	8.702669705	9.808612178	6.919411073
TCGA-IB-A6UG-01A-32R-A33R-07	7.608639157	8.675564205	7.310481535
TCGA-IB-A7LX-01A-12R-A36G-07	9.75336887	10.06823811	8.931359749
TCGA-IB-A7M4-01A-11R-A36G-07	8.721902982	8.688502267	7.93715809
TCGA-IB-AAUM-01A-11R-A37L-07	6.0674275	6.93716122	4.885396401
TCGA-IB-AAUN-01A-12R-A38C-07	8.788363557	9.567345369	8.337972302
TCGA-IB-AAUO-01A-12R-A38C-07	9.088703974	9.392122915	9.346926815
TCGA-IB-AAUP-01A-11R-A37L-07	7.617173925	9.229429436	7.458431673
TCGA-IB-AAUQ-01A-22R-A41I-07	8.586185779	9.421227169	8.260421075
TCGA-IB-AAUR-01A-21R-A38C-07	7.776340836	8.442955567	8.154494807
TCGA-IB-AAUS-01A-12R-A38C-07	8.35076438	8.990627917	8.463661086
TCGA-IB-AAUT-01A-11R-A37L-07	7.718546491	8.292200732	7.511427086
TCGA-IB-AAUU-01A-11R-A37L-07	8.916226658	9.667463432	7.763468236
TCGA-IB-AAUV-01A-11R-A38C-07	8.420343775	8.032624444	8.462972489
TCGA-IB-AAUW-01A-12R-A38C-07	7.598930692	8.050395152	5.371655875
TCGA-L1-A7W4-01A-12R-A36G-07	9.437144085	8.725784209	9.839826901
TCGA-LB-A7SX-01A-11R-A33R-07	7.142240017	8.476443954	6.029698182
TCGA-LB-A8F3-01A-11R-A36G-07	7.386033198	8.34285081	6.03415405
TCGA-LB-A9Q5-01A-11R-A39D-07	6.721509792	7.74475009	6.624035772
TCGA-M8-A5N4-01A-11R-A26U-07	9.240984944	8.760852021	8.538328987
TCGA-OE-A75W-01A-12R-A320-07	7.949531353	9.324306035	7.232929522
TCGA-PZ-A5RE-01A-11R-A320-07	8.684334394	9.381388326	7.759122172
TCGA-Q3-A5QY-01A-12R-A320-07	7.468469842	8.222584613	7.038101914
TCGA-Q3-AA2A-01A-11R-A37L-07	8.541643889	9.154741995	7.866554644
TCGA-RB-A7B8-01A-12R-A33R-07	7.895352022	9.086018681	7.562680697
TCGA-RB-AA9M-01A-11R-A39D-07	8.480818078	9.372447417	7.531327502
TCGA-RL-AAAS-01A-32R-A39D-07	7.709960331	8.555778638	6.558160847
TCGA-S4-A8RM-01A-11R-A37L-07	7.684710932	8.670810185	5.562765117
TCGA-S4-A8RO-01A-12R-A37L-07	8.372521121	9.427462559	9.163292011
TCGA-S4-A8RP-01A-11R-A36G-07	7.852247756	9.316143337	7.377940631
TCGA-US-A774-01A-21R-A320-07	7.905663112	8.932708564	8.197004269
TCGA-US-A776-01A-13R-A33R-07	8.020136418	7.442889675	3.590652132
TCGA-US-A779-01A-11R-A320-07	8.073585088	8.66792543	5.610934802
TCGA-US-A77E-01A-11R-A320-07	8.194653285	8.594604082	7.881241278
TCGA-US-A77G-01A-11R-A320-07	7.699809622	8.514701852	4.269734478
TCGA-US-A77J-01A-11R-A320-07	7.764450534	8.317762472	7.048171752
TCGA-XD-AAUG-01A-61R-A41B-07	7.922018906	8.420321906	8.106962697
TCGA-XD-AAUH-01A-42R-A41B-07	7.573210326	8.287853428	7.135773652
TCGA-XD-AAUI-01A-42R-A41B-07	8.72284612	9.074717003	7.381634346
TCGA-XD-AAUL-01A-21R-A39D-07	8.287951106	9.37988632	7.931926715
TCGA-XN-A8T3-01A-11R-A36G-07	7.971445113	9.262049858	8.707925627

TCGA-XN-A8T5-01A-12R-A36G-07	7.770621534	8.735265	6.863501381
TCGA-YB-A89D-01A-12R-A36G-07	8.691359625	9.425422393	8.165640969
TCGA-YH-A8SY-01A-11R-A37L-07	8.642971943	9.035533733	9.09316453
TCGA-YY-A8LH-01A-11R-A36G-07	9.023871539	10.0739228	5.609146324
TCGA-Z5-AAPL-01A-12R-A41B-07	8.141718445	8.355211428	6.40610983
