# Supplementary material for: Effects of anoxic prognostic model on immune microenvironment in pancreatic cancer
Source: Sci Rep. 2023 Jun 5;13:9104. doi: 10.1038/s41598-023-36413-9 (PMC10241784; doi:10.1038/s41598-023-36413-9)
Supplement: Supplementary file 4 — Supplementary Table S4. [file 41598_2023_36413_MOESM4_ESM.pdf]

Supplementary file 4：TABLE S4: Verification set from GSE85916 and ICGC-PACA-AU.

id	PKM	LDHA	PLAU
PDA_tumor_1	8.968392765	8.30195439	6.80088563
PDA_tumor_2	7.875946502	6.319328487	4.872044645
PDA_tumor_3	9.238470973	8.448954397	7.174455706
PDA_tumor_4	9.061167983	8.659772815	6.567267269
PDA_tumor_5	9.989014894	8.777267444	7.361118688
PDA_tumor_6	10.20915362	8.612285112	8.102662072
PDA_tumor_7	9.596517131	8.785686573	7.568931494
PDA_tumor_8	9.434003404	8.081462052	7.948127516
PDA_tumor_9	8.842838583	8.495117638	7.092946859
PDA_tumor_10	10.25879117	8.538640939	7.988546343
PDA_tumor_11	10.10221311	9.042060022	7.447108961
PDA_tumor_12	9.42006239	8.573639922	8.136141793
PDA_tumor_13	9.724218394	9.166981614	7.601108875
PDA_tumor_14	8.938704291	8.166594157	7.175958908
PDA_tumor_16	8.245827947	6.798433401	5.954488655
PDA_tumor_17	10.20444892	8.618407317	7.932476866
PDA_tumor_18	7.945246678	6.481654063	5.585254415
PDA_tumor_19	9.748118177	9.20177065	7.321348781
PDA_tumor_20	8.697296553	6.640055865	6.664944444
PDA_tumor_21	9.152249545	8.603821108	7.626971963
PDA_tumor_22	8.338385598	7.446203875	6.476505596
PDA_tumor_23	9.280742017	8.251554287	7.388939672
PDA_tumor_24	9.715853381	9.453751552	7.497482619
PDA_tumor_25	9.383473779	8.264882836	6.822057122
PDA_tumor_26	8.564590934	8.094425288	6.570639025
PDA_tumor_27	9.11067573	7.784206939	7.045466297
PDA_tumor_28	9.50392368	9.081576442	8.849760976
PDA_tumor_29	8.424925004	7.592668488	7.314866509
PDA_tumor_30	10.42606168	9.736188136	7.914573689
PDA_tumor_31	8.811271001	8.527204038	7.836627193
PDA_tumor_32	8.7381693	9.446032137	6.508258611
PDA_tumor_33	9.589291692	8.16320091	7.990125032
PDA_tumor_34	10.11641031	9.796581786	8.374564861
PDA_tumor_35	9.436471114	9.469606693	6.731200055
PDA_tumor_36	9.78764521	9.995785965	6.387514683
PDA_tumor_37	9.745804308	8.95684957	7.615993221
PDA_tumor_38	9.708875606	9.119195173	6.70706048
PDA_tumor_39	10.02977174	9.555380294	7.705351599
PDA_tumor_40	8.378029032	7.916244969	6.199791535
PDA_tumor_41	9.565693512	8.985250739	8.181183429
PDA_tumor_42	9.14111316	9.200575144	4.390855744

PDA_tumor_43	9.229754247	8.594404199	6.114642856
PDA_tumor_44	9.987956689	9.308563323	8.46972426
PDA_tumor_45	8.838699111	8.955189595	6.352221768
PDA_tumor_46	9.687582533	9.133672192	7.47886
PDA_tumor_47	8.360831225	7.479774487	6.168680601
PDA_tumor_48	10.27261602	9.802089707	7.718224165
PDA_tumor_49	10.46375294	10.32662462	8.159192269
PDA_tumor_50	8.959004355	8.103628034	6.470126908
PDA_tumor_51	9.127387763	8.020758388	8.076583004
PDA_tumor_52	9.809875946	9.066645025	7.821186488
PDA_tumor_53	10.21899577	9.841582055	8.669593639
PDA_tumor_54	10.3241643	9.802814552	7.759098633
PDA_tumor_55	10.0174052	8.771740096	7.508225055
PDA_tumor_56	8.49649266	7.636516056	5.959996309
PDA_tumor_57	9.703718023	9.300883975	7.485177148
PDA_tumor_58	9.290554353	9.309693748	7.933048016
PDA_tumor_59	10.16264478	9.884495986	7.610820794
PDA_tumor_60	10.37776415	9.853287012	8.855636179
PDA_tumor_61	9.717503899	9.870708788	7.28081442
PDA_tumor_62	9.458265015	9.643201339	7.698877756
PDA_tumor_63	9.288758744	8.334397305	7.05862196
PDA_tumor_64	9.97094348	10.25817474	8.028171328
PDA_tumor_65	9.601314392	8.858754008	8.653640305
PDA_tumor_66	10.04200807	9.570387802	8.443701299
PDA_tumor_67	8.954720355	7.960613921	7.486578882
PDA_tumor_68	9.64565979	9.258675397	7.400188828
PDA_tumor_69	10.34183419	10.54121041	7.90239879
PDA_tumor_70	9.326628236	8.400624998	7.391967284
PDA_tumor_71	8.936057767	8.204471389	6.992726138
PDA_tumor_72	9.903863164	10.02436571	8.706253174
PDA_tumor_73	9.420306754	8.624132255	7.037067585
PDA_tumor_74	9.787170911	8.609469789	7.841628212
PDA_tumor_75	10.56787424	9.344773803	9.154031645
PDA_tumor_76	10.05063868	9.62098738	7.9889967
PDA_tumor_77	9.859848835	9.885422672	8.262863042
PDA_tumor_78	9.588015084	9.179059829	7.877871653
PDA_tumor_79	9.652118285	9.65103136	8.194764135
PDA_tumor_80	9.960067315	8.985109317	7.644382252
ICGC_0006	7.667925252	7.631293715	3.981378883
ICGC_0007	9.325207473	8.807056746	6.635178658
ICGC_0009	9.687977014	8.687751958	5.960107626
ICGC_0020	9.139816672	8.530201387	6.004478948
ICGC_0021	9.73783477	9.39640661	7.446715944
ICGC_0025	10.12840533	8.891525498	7.763784599

ICGC_0026	9.948847022	9.811717849	7.027116121
ICGC_0031	9.977096669	9.164469242	6.618311218
ICGC_0033	8.301039249	8.815092645	5.120800938
ICGC_0037	9.31429652	9.950674053	8.483213145
ICGC_0048	8.724361972	8.082063268	4.971604746
ICGC_0051	9.748087983	9.062368018	6.415633231
ICGC_0052	8.287350441	8.26546888	7.462785362
ICGC_0053	9.331091696	9.260333184	7.970353849
ICGC_0054	9.87447188	9.313776744	6.603820773
ICGC_0055	9.771166875	9.208442842	8.033603334
ICGC_0059	8.969496791	8.321672325	4.958744199
ICGC_0061	9.312601604	9.915873101	4.693773135
ICGC_0063	8.090456001	9.282015046	3.513874597
ICGC_0066	9.537761103	9.103475708	5.313987538
ICGC_0067	9.510780493	10.08164001	7.590151104
ICGC_0075	8.839004526	8.403035472	4.320451411
ICGC_0087	9.631661522	8.696227566	7.319706503
ICGC_0088	10.2540199	9.266139095	7.151523033
ICGC_0099	9.465846684	8.266161043	7.672063604
ICGC_0103	8.896450219	8.136419552	4.056806996
ICGC_0105	9.369742026	8.329315071	5.293855609
ICGC_0108	9.128209842	9.529230037	7.397209978
ICGC_0109	9.614370805	8.450424103	5.445448363
ICGC_0114	10.27641072	10.3901564	7.819836983
ICGC_0115	10.05997706	9.670546795	6.465172261
ICGC_0124	8.752947799	8.610525681	6.094995165
ICGC_0134	9.384033767	9.459667714	6.825727308
ICGC_0135	9.545907072	9.294077132	7.046384207
ICGC_0139	9.652422029	8.489427914	4.054031148
ICGC_0140	9.701554512	8.621324978	5.15396714
ICGC_0141	10.77514085	9.831367718	7.855161654
ICGC_0143	9.27635267	8.09493031	6.989679357
ICGC_0144	8.874222257	8.168630924	6.244713324
ICGC_0146	9.575319	8.71989845	7.137328287
ICGC_0149	9.784546254	10.15102525	6.049628205
ICGC_0150	8.362972997	9.065246874	5.65188557
ICGC_0153	9.051393754	8.784313945	5.733465154
ICGC_0169	10.08031733	9.10333736	4.98929194
ICGC_0185	9.581386886	9.154793411	5.801577682
ICGC_0188	9.344019261	10.00283787	8.248032326
ICGC_0192	10.00144596	9.662468429	6.126451622
ICGC_0199	10.07991551	9.751380863	8.253429644
ICGC_0201	9.397318913	8.760075967	6.928110723
ICGC_0205	9.178244308	9.559346916	6.269163753

ICGC_0206	10.46282811	9.414329177	8.209599891
ICGC_0207	10.87843338	10.56027356	7.442901763
ICGC_0212	10.12658208	9.390024886	6.976334915
ICGC_0214	9.147167701	8.763471568	6.618558308
ICGC_0215	9.640638517	8.898303968	7.004321522
ICGC_0223	9.270876132	8.968455601	6.424295481
ICGC_0224	9.129465635	8.965237783	3.876093008
ICGC_0227	9.60701303	10.64776227	7.971807876
ICGC_0230	9.155416527	9.842085752	5.688699157
ICGC_0235	9.771755532	9.637211449	6.791396563
ICGC_0295	9.344087955	8.961408283	6.736423495
ICGC_0296	9.820199523	9.697858054	7.697527433
ICGC_0300	8.118187893	8.689432731	6.091693242
ICGC_0301	9.358421849	8.728910864	6.438835846
ICGC_0303	9.695652378	9.459313314	6.050509241
ICGC_0304	9.529556556	8.998544194	6.485101533
ICGC_0309	9.215163142	9.517483128	5.09474799
ICGC_0312	9.121340111	9.20522568	6.434495663
ICGC_0313	10.71012448	10.24938171	8.39273852
ICGC_0315	9.494338508	9.434419082	7.630372429
ICGC_0321	10.36808113	10.20893497	7.22717377
ICGC_0326	9.595961013	8.919515287	6.214627639
ICGC_0338	9.50862992	8.749505469	4.437403993
ICGC_0354	9.989842383	8.779495875	6.851260064
ICGC_0365	9.539147157	9.521222676	6.921285392
ICGC_0391	7.418930524	8.041002453	3.853115957
ICGC_0392	10.72570238	10.32038965	9.870123104
ICGC_0393	10.15637041	9.509739278	7.962031046
ICGC_0395	7.95438885	8.477368855	5.52898105
ICGC_0406	8.571102054	8.920089627	6.30511782
ICGC_0412	8.583199092	8.410717031	5.624814167
ICGC_0415	8.783130564	8.269838144	4.400454354
ICGC_0417	9.291735288	8.825614134	5.717014183
ICGC_0419	8.655849594	8.290341516	4.546849401
ICGC_0420	9.719376665	9.300756853	7.352297623
ICGC_0486	9.478032929	9.103200166	5.704831513
ICGC_0502	9.712482667	8.643342492	5.40152066
ICGC_0507	8.728673858	8.004291441	4.464442887
ICGC_0518	9.008192764	9.031526688	5.706214745
ICGC_0521	10.06232208	9.090496224	6.686561299
ICGC_0522	8.497351246	7.181952076	6.001364444
ICGC_0526	9.394478204	8.883511159	5.088909837
ICGC_0535	10.02934315	9.799254335	6.529342338
ICGC_0536	9.154086421	8.920754271	7.169861433

ICGC_0543	10.53543565	10.0857286	8.145501415
