# Supplementary figures and images for: Effects of anoxic prognostic model on immune microenvironment in pancreatic cancer
Source: Sci Rep. 2023 Jun 5;13:9104. doi: 10.1038/s41598-023-36413-9 (PMC10241784; doi:10.1038/s41598-023-36413-9)

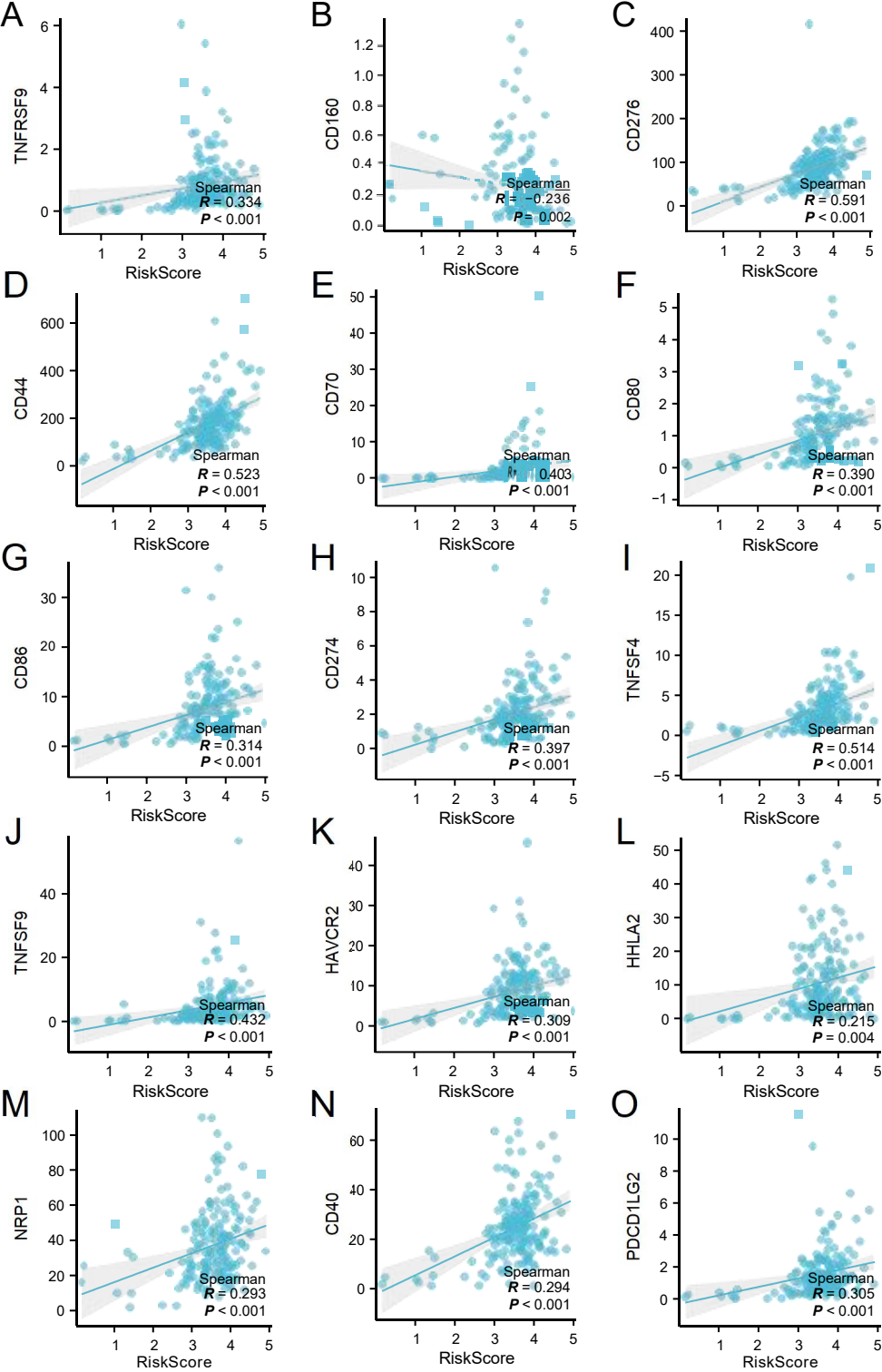

Supplementary file 5 : FIGURE S1: Scatter plot of Immune checkpoint associated with risk score

Supplement: Supplementary file 5 — Supplementary Figure S1. [file 41598_2023_36413_MOESM5_ESM.pdf]
